# Combined ACEI and ARB therapy and ICU mortality in critically ill COVID-19 patients: a retrospective cohort study

**DOI:** 10.3389/fphar.2026.1714530

**Published:** 2026-03-05

**Authors:** Ivanny Marchant, Gloria Balcazar, Valentina Pozo, Pablo Olivero, Belén Rodríguez, Romina Castillo, Hilda Espinoza

**Affiliations:** 1 Laboratorio de Modelamiento en Medicina, Facultad de Medicina, Universidad de Valparaíso, Valparaíso, Chile; 2 Unidad de Estudios Clínicos, Facultad de Medicina, Universidad de Valparaíso, Valparaíso, Chile; 3 Magister en Gestión Farmacéutica y Farmacia Asistencial, Escuela de Química y Farmacia, Universidad de Valparaíso, Valparaíso, Chile; 4 Unidad de Cuidados Intensivos, Hospital Dr. Gustavo Fricke, Viña del Mar, Chile; 5 Laboratorio de Estructura y Función Celular, Facultad de Medicina, Universidad de Valparaíso, Valparaíso, Chile; 6 Escuela de Química y Farmacia, Facultad de Farmacia, Universidad de Valparaíso, Valparaíso, Chile

**Keywords:** acid-base imbalance, angiotensin-converting enzyme inhibitors (ACEI), angiotensin receptor antagonists, COVID-19, intensive care unit, mortality

## Abstract

**Introduction:**

The clinical safety and potential benefits of renin–angiotensin–aldosterone system (RAAS) inhibitors in COVID-19 remain debated, particularly in critically ill populations. Evidence on combined angiotensin-converting enzyme inhibitor (ACEI) and angiotensin receptor blocker (ARB) therapy is limited, and the potential interaction with acid–base status has not been sufficiently explored.

**Methods:**

We conducted a retrospective cohort study including adults with PCR-confirmed COVID-19 admitted to the intensive care unit (ICU) at Hospital Dr. Gustavo Fricke (Chile) between March 2020 and December 2021. Patients were categorized according to RAAS therapy at admission (none, ACEI only, ARB only, ACEI + ARB) and arterial bicarbonate (HCO_3_^−^) levels (low <21 mEq/L, normal 21–27 mEq/L, high >27 mEq/L). Changes in HCO_3_^−^ during the first 48 h were evaluated. The primary outcome was in-hospital mortality; secondary outcomes included ICU length of stay and duration of mechanical ventilation. Group comparisons used chi-square and non-parametric tests.

**Results:**

Of 2,838 hospitalized patients, 671 required ICU admission and 655 had complete data for analysis. Overall ICU mortality was 34.2%. Combined ACEI + ARB therapy was associated with lower mortality (16.9%) compared with no RAAS blockade (38.3%; p < 0.05), whereas ACEI or ARB monotherapy showed no significant association. Among patients with low or normal admission HCO_3_^−^ levels, early increases within 48 h were associated with reduced mortality. Patients with elevated baseline HCO_3_^−^ who survived experienced longer ICU stays and prolonged mechanical ventilation.

**Discussion:**

In this observational ICU cohort, dual RAAS blockade was associated with lower in-hospital mortality, although causal inference is limited by the retrospective design and incomplete pharmacologic exposure data. Early bicarbonate increase may reflect renal adaptive capacity and has potential prognostic value. Prospective controlled studies are needed to clarify the clinical relevance of RAAS modulation and metabolic biomarkers in severe COVID-19.

## 1 Introduction

Since its emergence in 2019, severe acute respiratory syndrome coronavirus-2 (SARS-CoV-2) has had a huge global impact on public health, with more than seven million deaths worldwide on 17 August 2025, according to WHO data (COVID-19 cases WHO COVID-19 dashboard, 2021). Although most patients present mild symptoms, a subset of patients develop severe diseases requiring hospitalization, often progressing to acute respiratory distress syndrome (ARDS) and multiorgan failure. It is well-established that a range of comorbidities, including cardiovascular, renal, and pulmonary diseases, are significant risk factors for poorer outcomes. Early in the pandemic, a contentious issue emerged concerning the continuation of treatment with angiotensin-converting enzyme inhibitors and angiotensin receptor blockers in patients infected with SARS-CoV-2.This controversy arose due to the modulatory effect of these medications on angiotensin-converting enzyme 2 (ACE2) expression, which functions as the viral entry receptor (Di Castelnuovo et al., 2020; Zhang et al., 2021).

Mechanistically, ACEIs and ARBs reduce angiotensin II–mediated inflammation and fibrosis via modulation of the renin–angiotensin system. ACE2, which degrades angiotensin II into angiotensin (COVID-19 Cases, 2021; Di Castelnuovo et al., 2020; Zhang et al., 2021; Study Details, 2019; Lee et al., 2024; Ma et al., 2021; Zhang et al., 2020), has been demonstrated to exert anti-inflammatory and antifibrotic effects. Consequently, RAAS blockers may offer a safeguard against lung injury notwithstanding the theoretical concerns regarding heightened ACE2-mediated viral entry (Study Details Elimination or prolongation, 2019; Lee et al., 2024). Recent randomized trials and meta-analyses suggest that these agents do not worsen COVID-19 outcomes and may even confer benefits in certain populations (Ma et al., 2021; Zhang et al., 2020; Saavedra, 2020). However, most available evidence comes from general hospitalized cohorts rather than critically ill patients (Lopes et al., 2021; Baral et al., 2021), thus engendering a degree of uncertainty regarding the impact of said cohorts on intensive care unit populations. Furthermore, physiologic factors such as acid-base balance, which is recognized as a factor influencing prognosis in cases of severe COVID-19 (Alfano et al., 2022) have rarely been considered in relation to RAAS blockade.

Critically ill patients with COVID-19 frequently exhibit complex metabolic and physiologic derangements that extend beyond pulmonary pathology alone (Rahman et al., 2025). Acid–base disturbances are highly prevalent in these patients and reflect the interplay between respiratory dysfunction, systemic inflammation, tissue hypoxia, and renal compensatory mechanisms (Achanti and Szerlip, 2023; Sinatra et al., 2025). Arterial bicarbonate levels serve as an integrative marker of these processes, capturing information related to ventilatory failure and hypercapnia, metabolic acidosis driven by hypoperfusion and lactate accumulation, and the kidney’s capacity to maintain acid–base homeostasis during critical illness (Dominguez et al., 2025).

Emerging clinical and experimental evidence suggests that critically ill patients may display distinct phenotypic patterns based on acid–base status (Chiumello et al., 2025). Low bicarbonate states are commonly associated with metabolic acidosis, hyperinflammatory responses, impaired tissue oxygen utilization, and adverse outcomes (Peetermans et al., 2025). Conversely, elevated bicarbonate levels often reflect renal compensation for respiratory acidosis and may indicate advanced pulmonary dysfunction, prolonged hypoventilation, or evolving multiorgan failure (Sirol Aflah et al., 2025). These acid–base patterns should therefore be interpreted not as isolated biochemical abnormalities, but as dynamic phenotypic markers of disease severity, organ involvement, and adaptive capacity in severe COVID-19 (Peetermans et al., 2025).

The RAAS plays a central role not only in cardiovascular and inflammatory regulation, but also in renal acid–base handling, including bicarbonate reabsorption, ammoniagenesis, and tubular proton secretion (Dominguez et al., 2025). During critical illness, RAAS activation contributes to renal stress and maladaptive responses, while pharmacologic RAAS blockade may modulate these pathways and influence acid–base balance (Dominguez et al., 2025). In the context of SARS-CoV-2 infection, which is characterized by RAAS dysregulation, endothelial injury, and frequent kidney involvement, the interaction between RAAS inhibition and acid–base homeostasis may be clinically relevant yet remains insufficiently explored in ICU populations (Nadim et al., 2020).

The present study aimed to evaluate the association between ACEI and ARB use, either as monotherapy or in combination, and in-hospital mortality among critically ill patients with COVID-19. Additionally, we sought to determine whether arterial bicarbonate levels at ICU admission and their early trajectory during the first 48 h, as markers of acid–base status and renal adaptive response, modify this association and provide prognostic information in severe disease.

## 2 Methods

### 2.1 Study design and setting

A retrospective cohort study was conducted on adults with confirmed SARS-CoV-2 infection admitted to the intensive care unit at Hospital Dr. Gustavo Fricke (Viña del Mar, Chile) between March 2020 and December 2021. The study was approved by the Bioethics Committee of the Faculty of Medicine of the University of Valparaiso, and the requirement for informed consent was waived due to the retrospective nature of the analysis.

### 2.2 Participants

Participants meeting the specified criteria included adults (aged ≥18 years) with PCR-confirmed SARS-CoV-2 infection who required ICU admission. Patients presenting respiratory symptoms but diagnosed with non-COVID-19 etiology (e.g., bacterial pneumonia, pulmonary edema, or asthma exacerbation) were excluded from the study.

### 2.3 Exposure and grouping

**FIGURE 1:**
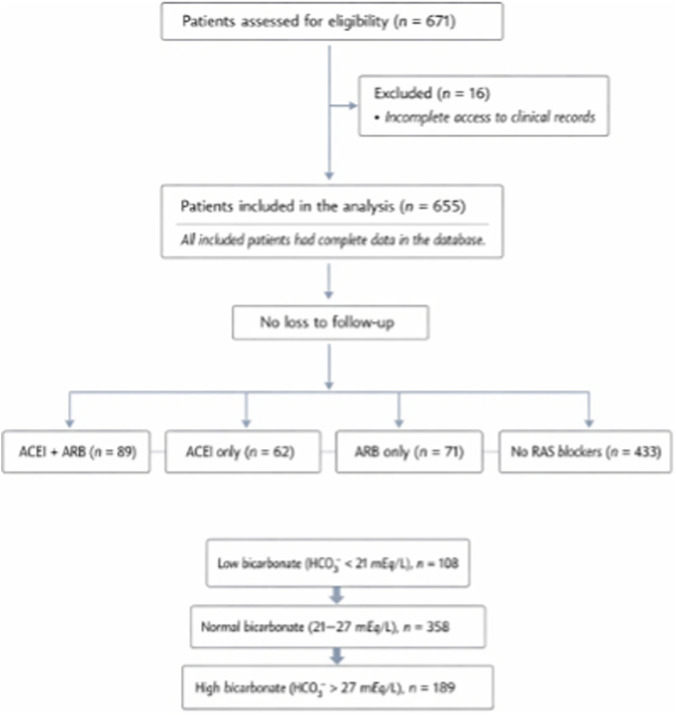
Study design. Flow diagram of the retrospective cohort study conducted at Hospital Dr. Gustavo Fricke (Viña del Mar, Chile) between March 2020 and December 2021. Adults (≥18 years) with PCR-confirmed SARS-CoV-2 infection requiring admission to an intensive care unit (ICU) were included in the study. Patients with non-COVID-19 respiratory diagnoses were excluded from the study. Patients were stratified according to their medication (none, ACEI, ARB, ACEI + ARB) and admission bicarbonate (HCO_3_^−^) levels (low <21 mEq/L, normal 21–27 mEq/L, high >27 mEq/L). A secondary stratification was considered, the focus of which was changes in HCO3- category between ICU day 1 and day 2. The primary outcome was in-hospital mortality; secondary outcomes were ICU length of stay and duration of mechanical ventilation.

Patients were stratified according to their utilization of ACEIs/ARBs at the time of ICU admission. Due to the absence of reliable data in the clinical registry concerning the dosage, duration, timing of prescription, or chronic use of ACEIs or ARBs prior to admission, patients could not be stratified according to their respective doses of ARB or ACEI. A categorization system was employed, resulting in the classification of subjects into four groups: users of ACEI only, ARBs only, ACEIs and ARBs, and those not using ACEs nor ARBs. Furthermore, patients were categorized according to their serum bicarbonate (HCO_3_-) levels at the time of admission to the ICU: low (<21 mEq/L), normal (21–27 mEq/L), or high (>27 mEq/L) (Figure 1). A secondary stratification was performed based onchanges in HCO_3_- levels between ICU day 1 and 2 (where levels increased in comparison to those which remained stable).

### 2.4 Outcomes

The primary outcome was in-hospital mortality, defined as death occurring at any time during the index hospitalization, regardless of ICU discharge status. Twenty-eight–day mortality was not evaluated. Secondary outcomes were ICU length of stay and duration of mechanical ventilation. Data regarding prone positioning and its duration were not systematically available in the clinical records and were therefore not included in the analysis. Concomitant therapies (corticosteroids, antivirals, antibiotics, and other treatments) were not incorporated in this analysis due to incomplete documentation.

### 2.5 Statistical analysis

Categorical variables were expressed as frequencies and percentages, and continuous variables as medians with interquartile ranges. Comparisons between groups were made using chi-square tests for categorical variables and non-parametric tests for continuous variables, as appropriate. Associations between ACEI/ARB use, acid–base status, and outcomes were explored using stratified analyses. A p value < 0.05 was considered statistically significant. To undertake a more profound investigation into the impact of ACEI/ARB therapy on the survival of critically ill patients and the potential correlations with enhanced renal adaptation, a multivariable logistic regression analysis was conducted. This analysis incorporated RAAS blockers and bicarbonate status within the ICU. Power calculation was not performed due to the retrospective design and inclusion of all eligible patients during the study period. The analyses were conducted using R software, version 4.5.2. (R Core Team, 2025).

## 3 Results

Of the 2838 patients who were hospitalized with PCR-confirmed SARS-CoV-2 infection, 671 (23.6%) required admission to the ICU. Of these patients, 655 individuals formed our cohort due to a lack of access to clinical files in the remaining 16 patients. The overall in-hospital mortality rate among patients treated in the ICU was 34.2% (Table 1).

**TABLE 1:** Characteristics of patients by blood bicarbonate levels.

Characteristic	Overall	High bicarbonate	Normal bicarbonate	Low bicarbonate
	n = 655	n = 189	n = 358	n = 108
Age (years)	60 (50.68)	59 (50.66)	59 (48.68)	63 (55.71)
Male, n (%)	398 (60.8)	123 (65.1)	213 (59.5)	62 (57.4)
BMI	29.9 (26.5; 33.2)	30.0 (27.1; 35.1)	29.6 (26.5; 33.3)	29.5 (25.9; 31.1)
EHT, n (%)	343 (52.4)	93 (49.2)	181 (50.6)	69 (63.9)
T2DM, n (%)	211 (32.2)	59 (31.2)	108 (30.2)	44 (40.7)
CVD, n (%)	79 (12.1)	23 (12.2)	39 (10.9)	17 (15.7)
Cancer, n (%)	52 (7.9)	17 (8.9)	24 (6.7)	11 (10.9)
Dyslipidemia, n (%)	48 (7.3)	15 (7.9)	23 (6.4)	10 (9.3)
Pulmonary disease, n (%)	63 (9.6)	18 (9.5)	37 (10.3)	8 (7.4)
CKD, n (%)	44 (6.7)	6 (3.2)	24 (6.7)	14 (12.9)
Smoking status, n (%)	48 (7.3)	21 (11.1)	22 (6.1)	5 (4.6)
Body temperature (°C)	36.8 (36.1; 37.0)	36.8 (36.2; 37.1)	36.8 (36.0; 37.0)	36.7 (36.0; 37.0)
Heart rate (bpm)	88.0 (78; 100)	85 (71; 95)	88 (78; 100)	90 (80; 101)
Respiratory rate (rpm)	24 (22; 27)	24 (22; 26)	24 (22; 28)	24 (21; 26)
SaO2%	95 (92.0; 97.4)	95 (92.0; 97.0)	95 (92.0; 98.0)	94 (91.0; 97.0)
PaCO2 (mmHg)	37.8 (32.8; 44.6)	43.0 (39.0; 51.4)	36.0 (32.9; 42.0)	31.5 (27.2; 37.3)
HCO3 (mEq/L)	24.5 (22.0; 27.0)	28.9 (27.6; 31.0)	24.0 (22.9; 25.0)	18.2 (15.1; 19.7)
pH	7.41 (7.35; 7.45)	7.43 (7.37; 7.46)	7.42 (7.37; 7.45)	7.35 (7.29; 7.40)
Potassium (mEq/L)	4.0 (3.6; 4.4)	3.9 (3.6; 4.3)	4.0 (3.6; 4.4)	4.2 (3.7; 4.6)
Creatinine (mg/dL)	0.8 (0.6; 1.3)	0.8 (0.6; 1.0)	0.8 (0.6; 1.2)	1.2 (0.9; 2.4)
Eosinophils (n/μL)	10.1 (0.0; 84.6)	14.2 (0.0; 99.4)	7.7 (0.0; 75.6)	14.5 (0.0; 116.5)
ACEI, n (%)	160 (24.4)	45 (23.8)	76 (21.2)	39 (36.1)
ARB, n (%)	151 (23.1)	51 (27)	69 (19.3)	31 (28.7)
Corticoids, n (%)	619 (94.5)	178 (94.1)	345 (96.4)	96 (88.8)
Deaths, n (%)	224 (34.2)	68 (35.9)	105 (29.3)	51 (47.2)
ICU days	17 (8; 28)	18 (10; 32)	16 (7; 26)	17 (7; 31)
MV days	9 (0; 18)	13 (0; 20)	8.5 (0; 17)	5.5 (0; 16.3)

Statistics: n (%) or median and interquartile range for non-normally distributed data. Abbreviations: BMI, body mass index; EHT, Essential hypertension; T2DM, Type 2 diabetes mellitus; CVD, cardiovascular disease; CKD, chronic kidney disease; SaO2, arterial oxygen saturation; PaCO2, arterial carbon dioxide pressure; HCO3, arterial bicarbonate. ACEI, angiotensin conversion enzyme inhibitors; ARB, angiotensin receptor blockers; ICU, Intensive care unit; MV, mechanical ventilation.

**FIGURE 2:**
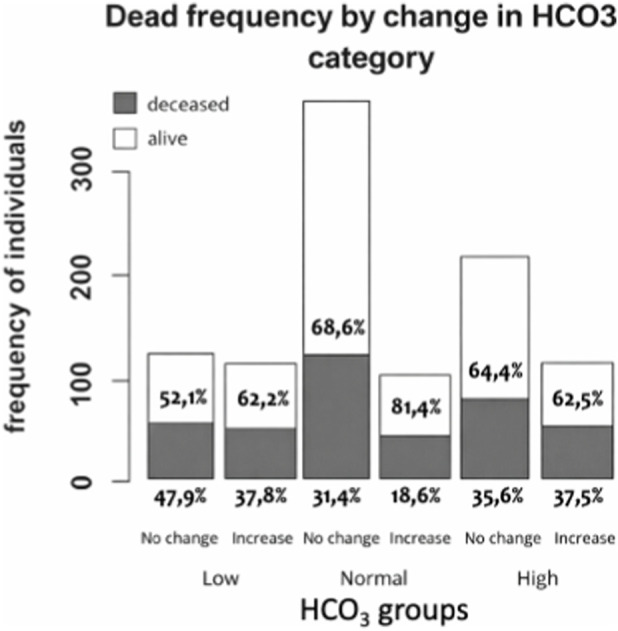
Relationship between HCO_3_^−^ levels and clinical outcomes. Boxplots show intensive care unit days (left) and mechanical ventilation days (right) for survivors and deceased patients. Patients are categorized into three groups based on their HCO_3_^−^ levels: low, normal, and high. Statistical comparisons were made using the Mann-Whitney U test, with significance indicated by p < 0.05 (*) and p < 0.005 (**). *Difference between high and low HCO3- groups and **difference between high and normal HCO3- groups.

When the study population was stratified according to RAAS inhibitor use, mortality was found to be significantly higher in patients not receiving RAAS blockers (38.3%) compared to those receiving combined ACEI + ARB therapy (16.9%, p < 0.05) (Table 2). No statistically significant differences in mortality were observed among patients receiving either ACEIs or ARBs as monotherapy. In the multivariable logistic regression model, combined ACEI + ARB therapy was independently associated with a significantly lower likelihood of the outcome, showing the strongest protective effect (OR 0.308, CI 95% 0.169–0.560, p < 0.001). ARB monotherapy demonstrated a modest negative association that did not reach statistical significance (p = 0.081), while ACEI monotherapy showed no meaningful effect (Table 3). Among patients in the low and normal HCO_3_^−^ groups, those with increasing HCO_3_-levels from ICU day 1 to day 2 exhibited significantly lower mortality compared with patients with stable levels (χ^2^ = 7.92, p = 0.019) (Figure 2). In contrast, no mortality differences were observed among patients with elevated admission HCO_3_-levels according to HCO_3_- trajectory during the first 48 h. In the multivariate analysis, higher bicarbonate levels at days 2 and 3 remained significantly independent predictors, both associated with reduced odds of the outcome (Table 3, p = 0.016 and p = 0.029, respectively).

**TABLE 2:** Mortality rate in patients positive for COVID-19 with or without ACEI and ARB drugs.

Treatment groups	Deceased n (%)	Survivors n (%)	Total
With ACEI and ARB, n (%)	15 (16.9)	74 (83.2)	89
Without ACEI nor ARB, n (%)	166 (38.3)	267 (61.7)	433
Total	181 (34.7)	341 (65.3)	522

**TABLE 3:** Impact of RAAS blockers and bicarbonate status by multivariable logistic regression analysis.

Variable	Estimate (β)	Std. Error	p-value	Odds Ratio (95% CI)
Intercept	1.66564	0.56353	0.0031 **	5.289 (1.753–15.961)
ACEI + ARB	–1.17921	0.30544	0.000113 ***	0.308 (0.169–0.560)
ARB only	–0.54284	0.31132	0.081218 (ns)	0.581 (0.316–1.070)
ACEI only	–0.07426	0.26887	0.78241 (ns)	0.928 (0.548–1.573)
HCO3_1	0.04856	0.02708	0.072927 (ns)	1.050 (0.995–1.107)
HCO3_2	–0.07510	0.03117	0.015974 *	0.928 (0.873–0.986)
HCO3_3	–0.06012	0.02758	0.029262 *	0.942 (0.892–0.994)

Significance codes: *** p < 0.001, ** p < 0.01, * p < 0.05, ns = not significant.

**FIGURE 3:**
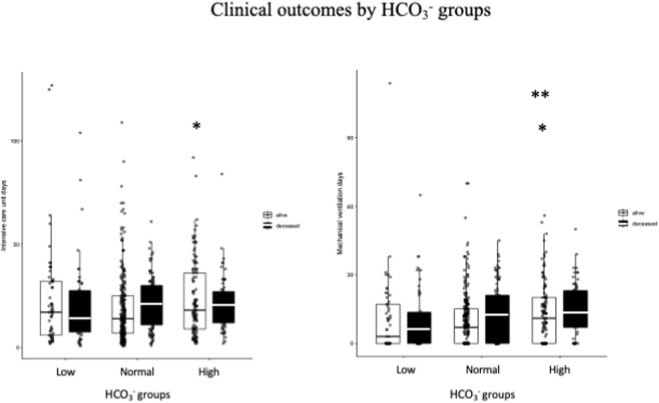
Comparison of HCO_3_^−^ levels in patients with low, normal, and high bicarbonate across different days of observation. Box plots display the distribution of arterial bicarbonate (HCO_3_^−^) levels across the three groups. White boxes represent survivors, and black boxes represent deceased patients. Comparisons were made using the Mann-Whitney U test due to the non-normal distribution of the main variable. Significant differences within groups across days are indicated by *p < 0.05 and **p < 0.005. Differences between survivors and deceased patients are marked by ^†^p < 0.05.

Analysis of secondary outcomes showed that survivors exhibiting elevated admission HCO_3_-levels required significantly extended ICU stays and prolonged mechanical ventilation in comparison to those with low or normal HCO_3_- levels (Figure 3). In particular, the median length of stay in the intensive care unit and the number of days spent on ventilation were found to be greater in the high HCO_3_-group (p < 0.05 and p < 0.005 respectively). This suggests that this subgroup may require a more protracted period of recovery.

## 4 Discussion

### 4.1 Main findings

**FIGURE 4:**
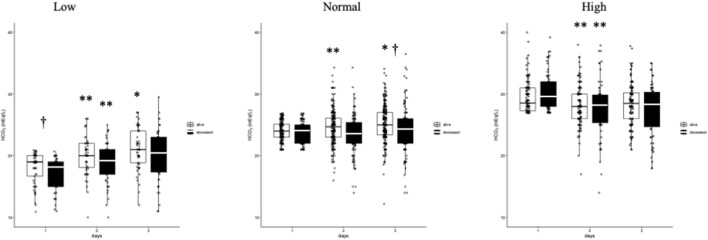
Relationship between changes in HCO_3_^−^ levels and mortality on the second day of ICU admission. This bar chart illustrates the proportion of survivors (white sections) and deceased individuals (grey sections) across different HCO_3_^−^ groups: low, normal, and high; each further subdivided based on whether their HCO_3_^−^ levels increased or remained stable over time. The chi-squared test result (χ^2^ = 7.9183, p = 0.01) indicates a statistically significant association between changes in HCO_3_^−^ levels and mortality.

In this retrospective cohort of critically ill COVID-19 patients, combined therapy with ACEIs and ARBs was associated with a statistically significant reduction of in-hospital mortality compared with patients not receiving RAAS inhibitors. Conversely, monotherapy with either ACEIs or ARBs alone showed no statistically significant effect on mortality, demonstrating only non-significant protective trends. A secondary observation of significance was that an early increase in arterial bicarbonate levels during the first 48 h of ICU admission was associated with enhanced survival, particularly among patients with initially low or normal values (Figure 4). This pattern suggests that bicarbonate kinetics may reflect underlying renal adaptive capacity rather than a direct pharmacological effect of RAAS inhibition. Collectively, these findings indicate that dual RAAS blockade identifies a subgroup with improved outcomes and underscore the prognostic value of dynamic acid–base monitoring in severe COVID-19.

### 4.2 Comparison with previous studies

The findings of this study are consistent with those of extensive observational studies and randomized trials demonstrating that ACEIs and ARBs are safe in COVID-19 patients and do not worsen clinical outcomes (Di Castelnuovo et al., 2020; Ma et al., 2021; Zhang et al., 2020; Lopes et al., 2021; Alfano et al., 2022; Biswas and Kali, 2021; Jardine et al., 2022; Kumar et al., 2022; Hippisley-Cox et al., 2020; Rizk et al., 2022). The BRACE CORONA and REPLACE COVID trials showed that continuation versus discontinuation of RAAS inhibitors did not significantly affect outcomes (Lopes et al., 2021; Cohen et al., 2021). Systematic reviews and meta-analyses have similarly reported neutral to modestly favorable effects, particularly in hypertensive and elderly populations (Di Castelnuovo et al., 2020; Lee et al., 2024; Lopes et al., 2021; Biswas and Kali, 2021; Rizk et al., 2022; Cohen et al., 2021).

However, mechanistic interpretations of benefit require caution. It has also been documented a reduction in viral load, lower IL-6 levels, and higher CD3/CD8 T-cell counts in patients receiving ACEI/ARB therapy, findings that have been associated with improved survival (Meng et al., 2020). Rather than implying a direct antiviral effect, these observations suggest that RAAS inhibition may modulate host inflammatory and immune responses during SARS-CoV-2 infection. Of particular significance is the observation that the SARS-CoV-2 has been found to downregulate the activity of the enzyme convertase, ACE2, thus reducing the production of the vasodilator peptide Ang-(1–7), and concomitantly enhancing the activity of the angiotensin II receptor, AT1R, which has been demonstrated to be deleterious to the cardiovascular system (Lopes et al., 2021; Cohen et al., 2021). While earlier hypotheses proposed that ACEI/ARB therapy increases ACE2 expression, current evidence does not support a clinically relevant effect on viral susceptibility. Instead, RAAS blockade may indirectly counterbalance angiotensin II–driven injury by attenuating AT1R signaling and favoring protective pathways (Verdecchia et al., 2020; Gheblawi et al., 2020; Zhu et al., 2020).

### 4.3 Mechanistic insights

ACE2, the viral entry receptor, is expressed in alveolar macrophages, type II pneumocytes, and airway epithelial cells (Katsi et al., 2021). In SARS-CoV-2 infection, viral entry involves Spike protein priming by TMPRSS2 or endosomal cathepsins following clathrin-mediated endocytosis (Inoue et al., 2007; Hoffmann et al., 2020; Jackson et al., 2021). This process drives ACE2 internalization and lysosomal degradation, reducing surface ACE2 expression and impairing angiotensin II conversion to angiotensin-(1–7) (Hoffmann et al., 2020).

The resulting accumulation of angiotensin II enhances AT1R-mediated pro-inflammatory, oxidative, and vasoconstrictive signaling, contributing to vascular injury and acute lung damage in severe COVID-19 (Verdecchia et al., 2020; Kuba et al., 2005). Dysregulation of this balance, characterized by excessive activation of the angiotensin II-AT1R axis, promotes hyperinflammation, oxidative stress, endothelial damage, and acute lung injury (Baral et al., 2021).

**FIGURE 5:**
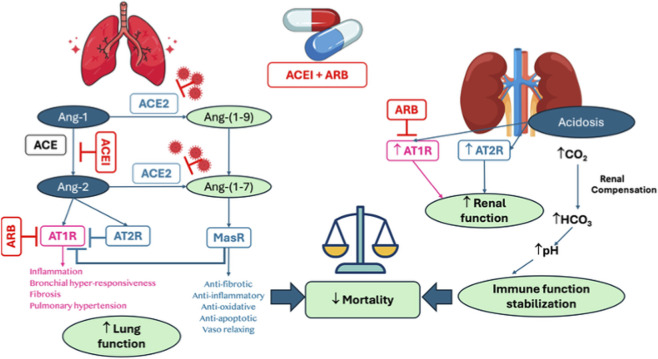
Schematic representation of the renin-angiotensin-aldosterone system and its effects on lung and renal function, acid-base balance, and immune stabilization. Angiotensin II (Ang-2) mediates inflammation, fibrosis, and bronchial hyper-responsiveness through AT1R activation. This process is counteracted by angiotensin receptor blockers (ARBs). ACE2 promotes the conversion of Ang-2 into Ang-(1-7), thereby exerting anti-inflammatory and anti-fibrotic effects via MasR. ARBs and ACE inhibitors (ACEIs) reduce the deleterious effects of Ang-2, thereby improving lung function. Acidosis, characterized by increased CO_2_ levels, triggers renal compensation through the production of HCO_3_^−^, thus restoring pH homeostasis and stabilizing immune function. These combined effects of these factors result in a reduction in mortality, attributable to improvements in both pulmonary and renal outcomes.

Clinical evidence has not supported the initial concern that ACEIs/ARBs worsen COVID-19 outcomes through ACE2 upregulation (Biswas and Kali, 2021; Jardine et al., 2022; Kumar et al., 2022; Hippisley-Cox et al., 2020). Instead, RAAS inhibition appears to restore the protective Ang-(1–7)/Mas axis and attenuate AT1R-mediated injury (Katsi et al., 2021). ARBs have also demonstrated protective effects in severe pneumonia, sepsis, and influenza (Katsi et al., 2021). Nevertheless, given the short-lived nature of angiotensin-(1–7), these mechanisms likely reflect signaling modulation rather than sustained vasodilatory effects. As illustrated in Figure 5, this dual blockade may not only attenuate the harmful Ang II–AT1R axis but also activates alternative lung- and kidney-protective pathways via AT2R and the Ang-(1–7)/Mas receptor axis. These mechanisms may be particularly relevant in the context of severe cases of COVID-19 and ARDS, where RAAS dysregulation contributes to pulmonary damage, endothelial dysfunction, and renal injury.

### 4.4 Renal regulation and acid–base balance

Metabolic acidosis is a frequent complication in critical illness, associated with catabolism, malnutrition, cardiovascular dysfunction, hormonal alterations, and progression of kidney disease (Zha and Qian, 2017; Wang et al., 2021; Podkowińska and Formanowicz, 2020; Kushwaha et al., 2024; Raphael, 2019). Experimental models show that acidosis induced by ammonium chloride (NH4Cl) activates intrarenal RAAS, increasing expression of ACE, angiotensinogen, angiotensin II, and ATR1/ATR2, leading to proteinuria and reduced glomerular filtration rate (Ng et al., 2011). Notably, overexpression of AT1R may persist even after serum bicarbonate levels normalization (Ng et al., 2011), indicating sustained renal stress.

Clinically, the discontinuation of ARBs has been associated with increased risk of acute kidney injury (AKI) in hypertensive COVID-19 patients (Soleimani et al., 2020), while low plasma bicarbonate levels independently predicts AKI (Kendrick et al., 2021). In our cohort study, early increases in bicarbonate were strongly associated with survival, suggesting preserved renal compensatory capacity rather than a direct hemodynamic effect of RAAS blockade.

Conversely, elevated admission HCO_3_^−^ likely reflects metabolic compensation for respiratory acidosis in patients with advanced pulmonary dysfunction (Xie et al., 2020; Gattinoni et al., 2020). Thus, admission bicarbonate levels should be interpreted as markers of disease severity, whereas bicarbonate kinetics during ICU stay may better reflect renal adaptive reserve.

### 4.5 Dual blockade versus treatment intensity effects

Combined ACEI + ARB therapy is rarely used in in contemporary practice and is generally restricted to selected patients with chronic kidney disease (CKD) and persistent proteinuria, diabetic nephropathy, or resistant hypertension, based on early assumptions that deeper RAAS inhibition might offer incremental renal protection (Düsing and Sellers, 2009; Doulton, 2006) Importantly, its effects in critically ill patients have not been systematically evaluated, and virtually no evidence exists regarding its impact in the context of acute viral pneumonia, ARDS, or COVID-19, underscoring the novelty of this study. This knowledge gap underlies the novelty of our study.

However, detailed information on drug dosage, treatment duration, and in-hospital modification were unavailable, precluding assessment of treatment intensity. Because of this limitation, the observed mortality association cannot be interpreted as evidence of a genuine synergistic effect. Instead, it may reflect differences in cumulative RAAS blockade intensity rather than the pharmacological interaction of combining an ACEI with an ARB. Recent evidence strongly supports this possibility. Contemporary hypertension and cardiorenal guidelines emphasize that optimized monotherapy - particularly titration to the maximal tolerated ARB dose - is preferred over dual blockade, which has consistently failed to improve outcomesand increases the risk of hyperkalemia, acute kidney injury, and symptomatic hypotension (Mancia et al., 2023; Cheung et al., 2021; Feng et al., 2019; Zhao et al., 2021; Insani et al., 2023; Pecoits-Filho et al., 2025). Dose–response effects of ARBs remain a key mechanism for cardiorenal protection without the incremental risk profile associated with combination therapy (Weir, 2007; Rastogi et al., 2011). Despite this, our findings show that neither ACEI nor ARB monotherapy was associated with a statistically significant reduction in mortality; whereas combined therapy demonstrated a robust association after comprehensive multivariable adjustment. This gradient raises the possibility of a true pharmacological interaction, whereby ARB-mediated ATR1 inhibition shifts signaling toward ATR2 pathways while ACE inhibitionlimits angiotensin II accumulation. Nonetheless, this interpretation remains speculative and must be approached with caution.

### 4.6 Strengths and limitations

Strengths of this study include a comprehensive real-world ICU cohort and sequential arterial blood gas measurements enabling dynamic evaluation of acid–base balance. Limitations include missing data on vaccination status, chronicity of therapy, drug dosage, adherence, and concurrent treatments. Dosage is particularly critical, as it influences both potential synergistic effects and adverse events such as hyperkalemia and GFR decline, which may affect renal bicarbonate handling. Accordingly, the association observed with dual ACEI + ARB therapy should be considered hypothesis-generating. Combined therapy should not be implemented clinically based on these findings alone. Prospective studies comparing dual blockade with maximally titrated monotherapy, incorporating detailed pharmacologic exposure and safety monitoring, are required. Bicarbonate kinetics, Ang-(1–7)/Ang II ratios, and renal biomarkers may facilitate more personalized therapeutic strategies.

### 4.7 Clinical implications and future directions

These findings underscore a potential association between dual RAAS blockade and improve outcomes in critically ill patients with severe respiratory infections, including SARS-CoV-2; however, this observation should be interpreted as hypothesis-generating rather than as evidence supporting clinical implementation.

Combined ACEI + ARB therapy remains discouraged in current clinical practice due to well-established risks of hyperkalemia, acute kidney injury, and reductions in glomerular filtration rate. Accordingly, our results should not be viewed as a justification for initiating or maintaining dual RAAS blockade in critically ill patients outside of a research setting.

The observed association between early increases in bicarbonate levels and survival highlights the value of continuous, dynamic acid–base monitoring as a crucial early prognostic marker, reflecting renal adaptive capacity rather than direct pharmacological effects of RAAS inhibition. Future randomized or carefully designed prospective studies should evaluate RAAS-modulating strategies in ICU populations, with particular emphasis on drug dosage, timing of exposure, renal safety endpoints, and metabolic phenotyping.

Rather than revisiting dual ACEI + ARB therapy as currently prescribed, future investigations may benefit from comparing optimized, maximally titrated ARB monotherapy against alternative RAAS-modifying approaches, including agents targeting other components of the RAAS cascade, such as neprilysin inhibitors or recombinant ACE2. The incorporation of integrative biomarkers - including bicarbonate kinetics, Ang-(1–7)/Ang II ratios, and longitudinal renal function parameters - may enable more personalized and mechanistically grounded therapeutic strategies in critically ill patients.

## 5 Conclusion

Combined therapy with ACEIs and ARBs in critically ill patients with severe acute respiratory syndrome coronavirus 2 (SARS-CoV-2) has been shown to result in a significantly lower in-hospital mortality rate when compared to patients not receiving RAAS blockers. Conversely, monotherapy showed no substantial impact on patient outcomes. Furthermore, an increase in serum bicarbonate levels during the initial 48 h of ICU admission has been demonstrated to be associated with an enhanced probability of survival, particularly among patients who initially exhibited low or normal bicarbonate levels. The findings suggest that dual RAAS blockade may exert a synergistic protective effect by modulating RAAS pathways and renal acid-base homeostasis. The results obtained demonstrate the continued efficacy of ACEIs and ARBs in critically ill patients with SARS-CoV-2 infection and underscore the necessity for prospective randomized trials to evaluate dual RAAS blockade and personalized strategies integrating metabolic and renal biomarkers. The present study provides novel evidence that dual RAAS blockade may confer survival benefits in severe cases of the disease, and highlights bicarbonate monitoring as a simple bedside tool for ICU prognosis.

## Data Availability

The raw data supporting the conclusions of this article will be made available by the authors, without undue reservation.

## References

[B1] Achanti A. Szerlip H. M. (2023). Acid-base disorders in the critically ill patient. Clin. J. Am. Soc. Nephrol. 18 (1), 102–112. 10.2215/CJN.04500422 35998977 PMC10101555

[B2] Alfano G. Fontana F. Mori G. Giaroni F. Ferrari A. Giovanella S. (2022). Acid base disorders in patients with COVID-19. Int. Urol. Nephrol. 54 (2), 405–410. 10.1007/s11255-021-02855-1 34115260 PMC8193956

[B3] Baral R. Tsampasian V. Debski M. Moran B. Garg P. Clark A. (2021). Association between renin-angiotensin-aldosterone system inhibitors and clinical outcomes in patients with COVID-19: a systematic review and meta-analysis. JAMA Netw. Open 4 (3), e213594. 10.1001/jamanetworkopen.2021.3594 33787911 PMC8013817

[B4] Biswas M. Kali M. S. K. (2021). Association of angiotensin-converting enzyme inhibitors and angiotensin-receptor blockers with risk of mortality, severity or SARS-CoV-2 test positivity in COVID-19 patients: meta-analysis. Sci. Rep. 11 (1), 5012. 10.1038/s41598-021-84678-9 33658619 PMC7930241

[B5] Cheung A. K. Chang T. I. Cushman W. C. Furth S. L. Hou F. F. Ix J. H. (2021). KDIGO 2021 clinical practice guideline for the management of blood pressure in chronic kidney disease. Kidney Int. 99 (3). 10.1016/j.kint.2020.11.003 33637192

[B6] Chiumello D. Aiello C. Pozzi T. Cressoni M. Brioni M. Algieri I. (2025). Effect of mannitol on diuresis and acid–base equilibrium in critically ill patients. ICMx 13 (1), 96. 10.1186/s40635-025-00807-y 40956364 PMC12440839

[B7] Cohen J. B. Hanff T. C. William P. Sweitzer N. Rosado-Santander N. R. Medina C. (2021). Continuation *versus* discontinuation of renin–angiotensin system inhibitors in patients admitted to hospital with COVID-19: a prospective, randomised, open-label trial. Lancet Respir. Med. 9 (3), 275–284. 10.1016/S2213-2600(20)30558-0 33422263 PMC7832152

[B8] COVID-19 cases WHO COVID-19 dashboard COVID-19 cases WHO COVID-19 dashboard (2021). Geneva: World Health Organization; Available online at: https://data.who.int/dashboards/covid19/cases (Accesed July 25, 2025).

[B9] Di Castelnuovo A. Costanzo S. Antinori A. Berselli N. Blandi L. Bonaccio M. (2020). RAAS inhibitors are not associated with mortality in COVID-19 patients: findings from an observational multicenter study in Italy and a meta-analysis of 19 studies. Vasc. Pharmacol. 10.1016/j.vph.2020.106805 PMC7521934 32992048

[B10] Dominguez R. J. A. Nyrup Odgaard L. Xue J. Nogueira Coelho J. Harris A. N. Thomas L. (2025). Sex differences in renal acid-base regulation. Am. J. Physiology-Renal Physiology 329 (5), F615–F626. 10.1152/ajprenal.00174.2024 PMC12667237 41015430

[B11] Doulton T. W. R. (2006). ACE inhibitor-angiotensin receptor blocker combinations: a Clinicians perspective. Mini-Reviews Med. Chem. 6 (5), 491–497. 10.2174/138955706776876168 16719821

[B12] Düsing R. Sellers F. (2009). ACE inhibitors, angiotensin receptor blockers and direct renin inhibitors in combination: a review of their role after the ONTARGET trial. Curr. Med. Res. Opin. 25 (9), 2287–2301. 10.1185/03007990903152045 19635044

[B13] Feng Y. Huang R. Kavanagh J. Li L. Zeng X. Li Y. (2019). Efficacy and safety of dual blockade of the renin-angiotensin-aldosterone system in diabetic kidney disease: a meta-analysis. Am. J. Cardiovasc Drugs 19 (3), 259–286. 10.1007/s40256-018-00321-5 30737754

[B14] Gattinoni L. Chiumello D. Caironi P. Busana M. Romitti F. Brazzi L. (2020). COVID-19 pneumonia: different respiratory treatments for different phenotypes? Intensive Care Med. 46 (6), 1099–1102. 10.1007/s00134-020-06033-2 32291463 PMC7154064

[B15] Gheblawi M. Wang K. Viveiros A. Nguyen Q. Zhong J. C. Turner A. J. (2020). Angiotensin-converting enzyme 2: SARS-coV-2 receptor and regulator of the renin-angiotensin system: celebrating the 20th anniversary of the discovery of ACE2. Circ. Res. 126 (10), 1456–1474. 10.1161/CIRCRESAHA.120.317015 32264791 PMC7188049

[B16] Hippisley-Cox J. Young D. Coupland C. Channon K. M. Tan P. S. Harrison D. A. (2020). Risk of severe COVID-19 disease with ACE inhibitors and angiotensin receptor blockers: cohort study including 8.3 million people. Heart 106 (19). 10.1136/heartjnl-2020-318314 32737124 PMC7509391

[B17] Hoffmann M. Kleine-Weber H. Schroeder S. Krüger N. Herrler T. Erichsen S. (2020). SARS-CoV-2 cell entry depends on ACE2 and TMPRSS2 and is blocked by a clinically proven protease inhibitor. Cell. 181 (2), 271–280.e8. 10.1016/j.cell.2020.02.052 32142651 PMC7102627

[B18] Inoue Y. Tanaka N. Tanaka Y. Inoue S. Morita K. Zhuang M. (2007). Clathrin-dependent entry of severe acute respiratory syndrome coronavirus into target cells expressing ACE2 with the cytoplasmic tail deleted. J. Virol. 81 (16), 8722–8729. 10.1128/JVI.00253-07 17522231 PMC1951348

[B19] Insani W. N. Whittlesea C. Ju C. Man K. K. Adesuyan M. Chapman S. (2023). Impact of ACEIs and ARBs-related adverse drug reaction on patients’ clinical outcomes: a cohort study in UK primary care. Br. J. Gen. Pract. 73, e832–e842. 10.3399/BJGP.2023.0153 37783509 PMC10563001

[B20] Jackson C. B. Farzan M. Chen B. Choe H. (2021). Mechanisms of SARS-CoV-2 entry into cells. Nat. Rev. Mol. Cell. Biol. 23 (1), 3–20. 10.1038/s41580-021-00418-x 34611326 PMC8491763

[B21] Jardine M. J. Kotwal S. S. Bassi A. Hockham C. Jones M. Wilcox A. (2022). Angiotensin receptor blockers for the treatment of covid-19: pragmatic, adaptive, multicentre, phase 3, randomised controlled trial. BMJ. 10.1136/bmj-2022-072175 36384746 PMC9667467

[B22] Katsi V. Pavlidis G. Charalambous G. Tousoulis D. Toutouzas K. (2021). COVID-19, angiotensin-converting enzyme 2 and renin-angiotensin system inhibition: implications for practice. Curr. Hypertens. Rev. 18 (1), 3–10. 10.2174/1573402117666210121100201 33475077

[B23] Kendrick J. Chonchol M. You Z. Jovanovich A. (2021). Lower serum bicarbonate is associated with an increased risk of acute kidney injury. J. Nephrol. 34 (2), 433–439. 10.1007/s40620-020-00747-8 32436182

[B24] Kuba K. Imai Y. Rao S. Gao H. Guo F. Guan B. (2005). A crucial role of angiotensin converting enzyme 2 (ACE2) in SARS coronavirus-induced lung injury. Nat. Med. 11 (8), 875–879. 10.1038/nm1267 16007097 PMC7095783

[B25] Kumar S. Nikravesh M. Chukwuemeka U. Randazzo M. Flores P. Choday P. (2022). Safety of ACEi and ARB in COVID‐19 management: a retrospective analysis. Clin. Cardiol. 45 (7), 759–766. 10.1002/clc.23836 35481554 PMC9110920

[B26] Kushwaha R. Vardhan P. S. Kushwaha P. P. (2024). Chronic kidney disease interplay with comorbidities and carbohydrate metabolism: a review. Life 14 (1), 13. 10.3390/life14010013 38276262 PMC10817500

[B27] Lee M. M. Y. Kondo T. Campbell R. T. Petrie M. C. Sattar N. Solomon S. D. (2024). Effects of renin–angiotensin system blockers on outcomes from COVID-19: a systematic review and meta-analysis of randomized controlled trials. Eur. Heart J. Cardiovasc Pharmacother. 10 (1), 68–80. 10.1093/ehjcvp/pvad067 37740450 PMC10766905

[B28] Lopes R. D. Macedo A. V. S. De Barros E. Moll-Bernardes R. J. Dos Santos T. M. Mazza L. (2021). Effect of discontinuing vs continuing angiotensin-converting enzyme inhibitors and angiotensin II receptor blockers on days alive and out of the hospital in patients admitted with COVID-19: a randomized clinical trial. JAMA - J. Am. Med. Assoc. 325 (3), 254–264. 10.1001/jama.2020.25864 PMC7816106 33464336

[B29] Ma J. Shi X. Yu J. Lv F. Wu J. Sheng X. (2021). Association of ACEi/ARB use and clinical outcomes of COVID-19 patients with hypertension. Front. Cardiovasc Med. 10.3389/fcvm.2021.577398 34136537 PMC8202940

[B30] Mancia G. Kreutz R. Brunström M. Burnier M. Grassi G. Januszewicz A. (2023). ESH guidelines for the management of arterial hypertension the task force for the management of arterial hypertension of the european society of hypertension: endorsed by the international society of hypertension (ISH) and the european renal associa. J. Hypertens. 41 (12), 1874–2071. 10.1097/HJH.0000000000003480 37345492

[B31] Meng J. Xiao G. Zhang J. He X. Ou M. Bi J. (2020). Renin-angiotensin system inhibitors improve the clinical outcomes of COVID-19 patients with hypertension. Emerg. Microbes Infect. 9 (1), 757–760. 10.1080/22221751.2020.1746200 32228222 PMC7170368

[B32] Nadim M. K. Forni L. G. Mehta R. L. Connor M. J. Liu K. D. Ostermann M. (2020). COVID-19-associated acute kidney injury: consensus report of the 25th acute disease quality initiative (ADQI) workgroup. Nat. Rev. Nephrol. 16, 747–764. 10.1038/s41581-020-00356-5 33060844 PMC7561246

[B33] Ng H. Y. Chen H. C. Tsai Y. C. Yang Y. K. Lee C. (2011). Activation of intrarenal renin-angiotensin system during metabolic acidosis. Am. J. Nephrol. 34 (1), 55–63. 10.1159/000328742 21659740

[B34] Pecoits-Filho R. Wong M. M. Y. Moorthy M. Banerjee D. Behera S. Calice-Silva V. (2025). Optimization of renin-angiotensin-aldosterone inhibitor therapies for evidence-based indications: a call to action from the cardio-kidney community. Am. J. Prev. Cardiol. 23, 100663. 10.1016/j.ajpc.2024.100663 40687934 PMC12275180

[B35] Peetermans M. Bohyn A. Meersseman P. Vanbastelaer S. Van Regenmortel N. Vanhonacker D. (2025). Outcomes of transplant recipients on ECMO for COVID-19 respiratory failure: an ELSO registry study. Crit. Care 29, 404. 10.1186/s13054-025-05636-9 40999467 PMC12465718

[B36] Podkowińska A. Formanowicz D. (2020). Chronic kidney disease as oxidative Stress- and inflammatory-mediated cardiovascular disease. Antioxidants 9 (8), 752. 10.3390/antiox9080752 32823917 PMC7463588

[B46] R Core Team (2025). R: a language and environment for statistical computing. Austria: R Foundation for Statistical Computing. Available online at: https://www.R-project.org/ (Accessed July 25, 2025 -January 31, 2026).

[B37] Rahman M. Ahmed S. Chakrabortty R. Selim S. Islam S. Rahman M. (2025). Metabolic parameters of Post-COVID patients in a tertiary care hospital in Bangladesh. J. Med. 26 (2), 124–130. 10.3329/jom.v26i2.84359

[B38] Raphael K. L. (2019). Metabolic acidosis in CKD: Core curriculum 2019. Am. J. Kidney Dis. 74 (2), 263–275. 10.1053/j.ajkd.2019.01.036 31036389

[B39] Rastogi A. Rashid M. Wright R. F. (2011). Reducing cardiorenal risk through combination therapy with a direct renin inhibitor. J. Clin. Hypertens. (Greenwich) 13 (11), 848–855. 10.1111/j.1751-7176.2011.00536.x 22051431 PMC8108923

[B40] Rizk J. G. Wenziger C. Tran D. Hashemi L. Hamid M. Elani S. (2022). Angiotensin-Converting Enzyme Inhibitor Angiotensin Recept. Blocker Use Assoc. Reduc. Mortal. Other Dis. Outcomes U. S. Veterans COVID-19. Angiotensin-converting enzyme inhibitor and angiotensin receptor blocker use associated with reduced mortality and other disease outcomes in US veterans with COVID-19, 82:43–54. 10.1007/s40265-021-01639-2 34914085 PMC8675115

[B41] Saavedra J. M. (2020). COVID-19, angiotensin receptor blockers, and the brain. Cell. Mol. Neurobiol. 40 (5), 667–674. 10.1007/s10571-020-00861-y 32385549 PMC7207082

[B42] Sinatra N. Cuttone G. Geraci G. Carollo C. Fici M. Senussi Testa T. (2025). Correlation between hypophosphatemia and hyperventilation in critically ill patients: causes, clinical manifestations, and management strategies. Biomedicines 28 (10), 2382. 10.3390/biomedicines13102382 41153669 PMC12561391

[B43] Sirol Aflah S. S. Kamal M. Ab Hamid N. Amran A. B. Lui S. C. Mohd Marzuki N. (2025). Respiratory and functional outcomes among severe COVID-19 infection survivors: a prospective observational study. Monaldi Arch. Chest Dis. 10.4081/monaldi.2025.3499 40994410

[B44] Soleimani A. Kazemian S. Karbalai Saleh S. Aminorroaya A. Shajari Z. Hadadi A. (2020). Effects of angiotensin receptor blockers (ARBs) on In-Hospital outcomes of patients with hypertension and confirmed or clinically suspected COVID-19. Am. J. Hypertens. 33 (12), 1102–1111. 10.1093/ajh/hpaa149 32920644 PMC7543264

[B45] Study Details Elimination or prolongation (2019). Elimination or prolongation of ACE inhibitors and ARB in coronavirus disease 2019 clinicalTrials.gov. Available online at: https://clinicaltrials.gov/study/NCT04338009#publications (Accessed August 26, 2025).

[B47] Verdecchia P. Cavallini C. Spanevello A. Angeli F. (2020). The pivotal link between ACE2 deficiency and SARS-CoV-2 infection. Eur. J. Intern Med. 76, 14–20. 10.1016/j.ejim.2020.04.037 32336612 PMC7167588

[B48] Wang X. H. Mitch W. E. Price S. R. (2021). Pathophysiological mechanisms leading to muscle loss in chronic kidney disease. Nat. Rev. Nephrol. 18 (3), 138–152. 10.1038/s41581-021-00498-0 34750550

[B49] Weir M. R. (2007). Angiotensin II receptor blockers: the importance of dose in cardiovascular and renal risk reduction. J. Clin. Hypertens. 6 (6), 315–325. 10.1111/j.1524-6175.2006.03473.x 15187494 PMC8109693

[B50] Xie J. Covassin N. Fan Z. Singh P. Gao W. Li G. (2020). Association between hypoxemia and mortality in patients with COVID-19. Mayo Clin. Proc. 95 (6), 1138–1147. 10.1016/j.mayocp.2020.04.006 32376101 PMC7151468

[B51] Zha Y. Qian Q. (2017). Protein nutrition and malnutrition in CKD and ESRD. Nutrients 9 (3), 208. 10.3390/nu9030208 28264439 PMC5372871

[B52] Zhang P. Zhu L. Cai J. Lei F. Qin J. J. Xie J. (2020). Association of inpatient use of angiotensin-converting enzyme inhibitors and angiotensin II receptor blockers with mortality among patients with hypertension hospitalized with COVID-19. Circ. Res. 126 (12), 1671–1681. 10.1161/CIRCRESAHA.120.317134 32302265 PMC7265882

[B53] Zhang Y. Sha T. Wu F. Hu H. Chen Z. Li H. (2021). Hypertension in patients hospitalized with COVID-19 in wuhan, China: a single-center retrospective observational study. Int. Heart J. 62 (2), 337–343. 10.1536/ihj.20-323 33678794

[B54] Zhao M. Qu H. Wang R. Yu Y. Chang M. Ma S. (2021). Efficacy and safety of dual vs single renin-angiotensin-aldosterone system blockade in chronic kidney disease: an updated meta-analysis of randomized controlled trials. Medicine 100 (35), E26544. 10.1097/MD.0000000000026544 34477114 PMC8415955

[B55] Zhu N. Zhang D. Wang W. Li X. Yang B. Song J. (2020). A novel coronavirus from patients with pneumonia in China, 2019. N. Engl. J. Med. 382 (8), 727–733. 10.1056/NEJMoa2001017 31978945 PMC7092803

