# Correction: Combined ACEI and ARB therapy and ICU mortality in critically ill COVID-19 patients: a retrospective cohort study

**DOI:** 10.3389/fphar.2026.1875271

**Published:** 2026-05-19

**Authors:** Ivanny Marchant, Gloria Balcazar, Valentina Pozo, Pablo Olivero, Belén Rodríguez, Romina Castillo, Hilda Espinoza

**Affiliations:** 1 Laboratorio de Modelamiento en Medicina, Facultad de Medicina, Universidad de Valparaíso, Valparaíso, Chile; 2 Unidad de Estudios Clínicos, Facultad de Medicina, Universidad de Valparaíso, Valparaíso, Chile; 3 Magister en Gestión Farmacéutica y Farmacia Asistencial, Escuela de Química y Farmacia, Universidad de Valparaíso, Valparaíso, Chile; 4 Unidad de Cuidados Intensivos, Hospital Dr. Gustavo Fricke, Viña del Mar, Chile; 5 Laboratorio de Estructura y Función Celular, Facultad de Medicina, Universidad de Valparaíso, Valparaíso, Chile; 6 Escuela de Química y Farmacia, Facultad de Farmacia, Universidad de Valparaíso, Valparaíso, Chile

**Keywords:** acid-base imbalance, angiotensin receptor antagonists, angiotensin-converting enzyme inhibitors (ACEI), COVID-19, intensive care unit, mortality

Affiliation “Magister en Gestión Farmacéutica y Farmacia Asistencial, Escuela de Química y Farmacia, Universidad de Valparaíso, Valparaíso, Chile” was omitted for authors **Ivanny Marchant, Pablo Olivero, Romina Castillo, and Hilda Espinoza**. This affiliation has now been added as the new Affiliation 3 for authors **Ivanny Marchant, Pablo Olivero, Romina Castillo, and Hilda Espinoza**. The remaining affiliations have been renumbered as 4, 5, and 6.

An incorrect Funding statement was provided. The funder “School of Chemistry and Pharmacy” was erroneously omitted from the statement. The correct funding statement reads:

“The author(s) declared that financial support was received for this work and/or its publication. This publication received financial support from the publication support fund of the School of Medicine and the School of Chemistry and Pharmacy at the University of Valparaíso.”

Two keywords were incorrect in the published article. “Angiotensinreceptor antagonists” was corrected to “angiotensin receptor antagonists”; “angiotensin-converting enzyme inhibitor” was corrected to “angiotensin-converting enzyme inhibitors (ACEI)”.

The original article has been updated.

